# Functional exercise training in persons with multiple sclerosis: a systematic review

**DOI:** 10.1007/s00415-025-13311-w

**Published:** 2025-08-23

**Authors:** Frederike Adammek, Weronika Gralla, Marie Kupjetz, Annette Rademacher, Philipp Zimmer, Eduard Isenmann, Niklas Joisten

**Affiliations:** 1 Research Group “Sports Medicine”, Institute for Sport and Sport Science, TU Dortmund University, 44227 Dortmund, Germany; 2 Department of Fitness and Health, IST University of Applied Sciences, 40233 Düsseldorf, Germany; 3 Asklepios MVZ Bayern GmbH, 93413 Cham, Germany; 4 Department of Neurology, Clinics of Valens, Rehabilitation Centre Valens, 7317 Valens, Switzerland; 5 Josef-Hospital, 44791 Bochum, Germany

**Keywords:** Exercise, Functional exercise, Rehabilitation, Functional training

## Abstract

Functional exercise training focuses on enhancing the body’s capacity for everyday movements by targeting a combination of strength, stability, balance, coordination, and flexibility. More flexible and less standardized exercise approaches, such as functional exercise training, may offer further benefits on improving symptoms in persons with MS (pwMS), but they have been less investigated. This review aims to provide a comprehensive overview of the existing literature on the effects of functional exercise training on functional outcomes (mobility, walking capacity, balance, and proprioception), physical (strength and endurance) and physiological performance measures, as well as patient-reported outcomes (Quality of Life, Fatigue) in pwMS. The systematic review was conducted according to the PRISMA guidelines. Literature searches were conducted in Medline (PubMed) and Cochrane CENTRAL (inception to 19th February 2025). We included studies investigating the effects of functional exercise training on any outcome in adult pwMS. The quality and reporting were evaluated using the Tool for the assEssment of Study qualiTy and reporting in EXercise (TESTEX). A total of 19 studies were included in the review, ranging from smaller pilot studies to larger randomized controlled trials. Interventions lasted between six and 16 weeks. Results of the studies included in this review were highly heterogeneous. Some of the included studies have demonstrated improvements in mobility, short and long walking capacity, balance, strength, and endurance measures as well as on quality of life and fatigue. Adherence to training was poorly reported. Functional exercise training for pwMS is a comprehensive approach to achieving functional improvements, strength gains, and improved endurance simultaneously, thereby promoting beneficial health effects. In future, a clear definition of functional exercise training is required, research should focus on the consistent reporting of treatment adherence, and investigate the long-term effects of functional exercise training in pwMS.

*PROSPERO registration number:* CRD42023448077.

**Supplementary Information:**

The online version contains supplementary material available at 10.1007/s00415-025-13311-w.

## Introduction

Multiple sclerosis (MS) is an inflammatory and neurodegenerative disease of the central nervous system that remains incurable despite substantial progress in the development and availability of effective pharmacological treatments [1, 2]. MS is characterized by heterogeneous symptoms that vary in their combination and severity and, for example, include walking impairment, reductions in muscle strength and cardiorespiratory fitness, and balance difficulties. In order to improve these symptoms, physical exercise has been established as an important non-pharmacological treatment adjunct over the past decades [3, 4]. Various exercise modalities have been shown to effectively reduce numerous MS symptoms, improve physical function, and have beneficial effects on depressive symptoms, fatigue, and health-related quality of life [3, 5–7]. Some of the beneficial effects of exercise seem to be generic, as, for example, suggested by a head-to-head meta-analysis that demonstrated comparable effects of strength and endurance training on walking impairment [8]. Some other beneficial effects are likely a result of modality-specific challenges to distinct motor or sensory systems (e.g., vestibular challenges) [9]. Accordingly, pwMS are advised to follow multimodal training schedules to maximize benefits [10]. PwMS, however, face considerable barriers to exercise engagement, that among others, are related to the MS-related disability, fatigue, and logistical issues (e.g., limited accessibility of suitable training facilities) [11]. Functional exercise training, which does not require large equipment or gym access, and simultaneously targets strength, stability, balance, coordination, and/or flexibility (Boyle 2016), may therefore qualify as a particularly time-efficient, low-cost, and easily accessible training option for pwMS, that is expected to have multidimensional beneficial effects for pwMS. Several systematic reviews have examined the effects of exercise interventions in pwMS, including those by Gunn et al. [12], Bae et al. [13], and Andreu-Caravaca et al. [14]. However, the focus of these reviews was on investigating the efficacy of different exercise interventions on functional outcomes such as balance, falls, fatigue, and strength, categorizing their interventions by modality (e.g., aerobic, resistance, combined training), setting (e.g., home-based, supervised), intensity (e.g., high-intensity interval training), and not investigating functional exercise training as intervention. To the authors’ knowledge, the effects of functional exercise training have not been evaluated systematically when focusing in particular on the intervention level itself. Therefore, this systematic review aimed to summarize the current evidence on the effects of functional exercise training in adult pwMS.

## Methods

This systematic review was prospectively registered in PROSPERO (identifier: CRD42023448077) on August 6, 2023 and conducted according to the Preferred Reporting Items for Systematic Reviews and Meta-Analyses (PRISMA) checklist for systematic reviews [15].

### Eligibility criteria

Eligibility criteria were defined using the Population, Intervention, Comparison, Outcomes and Study design Criteria (PICOS) [16].

#### Population

Studies investigating functional exercise training in adult pwMS (≥ 18 years) were included. Inclusion was not restricted to a specific disability range or MS phenotype.

#### Intervention and comparison

Functional exercise training was operationalized according to the definition of Boyle and regarded as exercise program mainly focused on the structured and goal-directed performance of exercises that are related to real-life activities (e.g., chair rise), involve multiple muscle groups simultaneously, primarily utilize closed-chain movements, are typically performed while standing and without relying on machines, and may use small equipment, such as balance pads or resistance bands, aside the person’s own body weight [17].

Based on this definition of functional training used in this review, studies that investigated any type of machine- or technology-assisted training, including e-Training, were excluded. Studies that combined exercise with non-exercise interventions (e.g., cognitive training, pharmacological treatment), conducted interventions that required certification (e.g., Pilates, mirror therapy), or performed isolated endurance, walking, balance, flexibility, or respiratory training interventions were also excluded. We further excluded studies investigating aquatic exercise and concepts of “task-oriented (circuit) training”. Task-oriented circuit training was excluded because it prioritizes the execution of tasks over movement quality, which does not align with the movement-based definition of functional training used in this review. There were no restrictions on comparators, if any, or outcomes.

#### Outcomes

There were no restrictions on outcome domains.

#### Study design

We included randomized controlled trials (RCTs), non-randomized controlled trials (CTs), and uncontrolled clinical trials (UCTs). Other article types (e.g., reviews, opinion articles, conference abstracts) were excluded. We considered peer-reviewed articles, published in English or German language.

### Information sources and search strategy

MK and WG independently conducted the literature search in Medline (PubMed) and the Cochrane Central Register of Controlled Trials (CENTRAL) (database inception to 19th February 2025). Each database was searched for relevant publications using a search string with keywords combined an MS search component and an intervention search component as follows: (((“Multiple Sclerosis”[Mesh]) OR (“multiple sclerosis”)) OR (MS)) AND (((((((((((((((((((“Circuit-Based Exercise”[Mesh]) OR (“Plyometric Exercise”[Mesh])) OR (“Resistance Training”[Mesh])) OR (“functional training”)) OR (“functional exercise*”)) OR (“combined training”)) OR (“combined exercise*”)) OR (“whole body training”)) OR (“whole body exercise*”)) OR (“circuit-based training”)) OR (“circuit-based exercise*”)) OR (“plyometric training”)) OR (“plyometric exercise*”)) OR (“resistance training”)) OR (“resistance exercise*”)) OR (“weight training”)) OR (“weight exercise*”)) OR (“strength training”)) OR (“strength exercise*”)). The initial search string was drafted for PubMed using Medical Subject Headings (MeSH) terms and (truncated) free search terms.

### Selection process

Automatic deduplication was performed using Citavi software (version 6.19 for Windows). Two independent investigators (WG and MK) performed a separate screening of titles and abstracts. Differences were discussed to reach a consensus by the two investigators. If they could not reach a consensus, a third investigator (NJ) was consulted. The same procedure was used for the screening of full texts.

### Data collection process, synthesis methods, and data items

Data extraction and synthesis was independently performed by MK and WG using customized templates and included data on study design, demographic and MS-specific characteristics of participants, intervention design, outcomes, and significant within- and between-group differences. We extracted quantitative outcomes, both subjective and objective, related to MS symptoms, performance, and mental health. We did not extract feasibility outcomes, qualitative measures, or goal attainment-related scores. Data extraction was validated by AR.

### Assessment of study quality, reporting, and risk of bias

Study quality and reporting were evaluated using the TESTEX tool (Table 1). Each study was graded and given 0–15 points based on specific criteria related to study quality (items one to five) and study reporting (items six to twelve) [18]. Study quality and reporting were independently assessed by two reviewers (MK and AR). In case of discrepancies, NJ was consulted to find consensus. The Cochrane Risk of Bias (RoB 2) tool was additionally used to evaluate all included studies [19]. No study was excluded based on the assessment results but the evaluation was taken into account during the interpretation of the synthesized data.

**Table 1:** Study quality and reporting of exercise training studies

Study	Study quality					Study reporting										Total
	1	2	3	4	5	6.1	6.2	6.3	7	8.1	8.2	9	10	11	12	
Abbaspoor et al. 2020	1	0	0	1	1	0	0	0	0	1	1	1	0	1	1	**8**
Akbar et al. 2020	1	0	0	1	1	0	0	0	0	1	1	1	0	1	0	**7**
Ayán Pérez et al. 2007	0	0	0	0	0	0	0	1	0	0	0	1	0	0	0	**2**
Bilek et al. 2022	1	1	0	1	1	1	0	0	0	1	1	1	0	1	0	**9**
Cakt et al. 2010	1	1	1	1	1	0	1	1	0	1	1	1	0	1	1	**12**
Coote et al. 2015	1	1	1	1	1	0	1	1	0	1	1	1	0	1	1	**12**
Correale et al. 2021	1	0	0	1	1	1	0	1	0	1	1	1	0	1	1	**10**
DeBolt and McCubbin 2004	1	1	0	1	0	1	0	1	0	1	1	1	0	1	1	**10**
Frevel and Mäurer 2015	1	1	0	1	0	1	0	0	0	1	1	1	0	1	1	**9**
Garret et al. 2013	1	1	1	1	1	0	0	1	0	1	1	1	0	1	1	**11**
Hosseini et al. 2018	1	1	0	0	0	1	0	0	0	1	1	0	0	1	1	**7**
Learmonth et al. 2012	1	1	0	1	1	1	0	1	1	1	1	1	0	1	0	**11**
Mardaniyan Ghahfarrokhi et al. 2022	1	1	1	1	1	1	1	1	1	1	1	1	0	1	1	**14**
Moghadasi et al. 2020	1	0	0	1	1	0	0	0	0	1	1	1	0	1	0	**7**
Motl. et al. 2012	1	0	0	0	0	1	0	1	0	0	0	1	0	1	1	**6**
Najafi et al. 2019	1	0	0	1	0	1	0	0	0	1	1	1	0	1	0	**7**
Sabapathy et al. 2011	0	1	0	0	0	0	1	1	0	1	1	1	0	1	1	**8**
Sosnoff et al. 2014	1	1	1	1	1	0	1	1	0	1	1	1	0	1	0	**11**
Zaenker et al. 2018	1	0	0	0	0	1	0	0	0	0	0	1	0	0	0	**3**
Total (across sub-scores)	17	11	5	14	11	10	5	11	2	16	16	18	0	17	11	**Ø 8.63**

Study quality items: 1. Eligibility criteria specified; 2. Randomization specified; 3. Allocation concealment; 4. Groups similar at baseline; 5. Blinding of assessor. Study reporting items: 6.1 outcome measures assessed in 85% of participants; 6.2 Reporting of adverse events; 6.3 Reporting of exercise attendance; 7. Intention-to-treat analysis; 8.1 Between-group statistical comparisons reported (primary outcome); 8.2 Between-group statistical comparisons reported (secondary outcome); 9. Point measures and measures of variability; 10. Activity monitoring in control groups; 11. Relative exercise intensity remained constant; 12. Exercise volume characteristics and energy expenditure. Criterion fulfilled = 1; Criterion not fulfilled = 0. Bold = total score

## Results

**Fig. 1:**
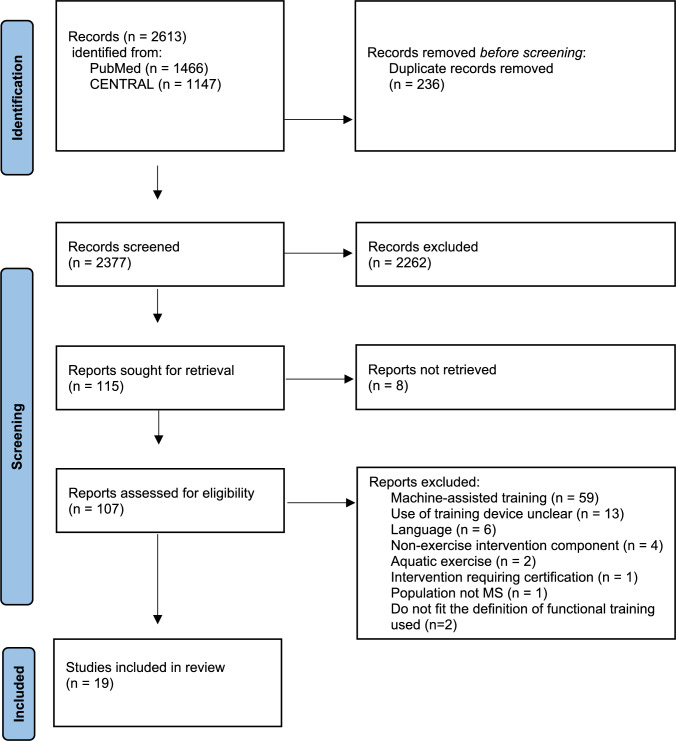
PRISMA flowchart. *Modified acc. to Page *et al*. 2021. CENTRAL* Cochrane central register of controlled trials, *MS* multiple sclerosis

Results of the database search and study selection process are presented in Fig. 1. The search strategy yielded 2613 hits. After deduplication, 2377 abstracts were screened for eligibility. We retrieved 115 full texts, among which *N* = 21 articles met the eligibility criteria and were included in this review.

### Characteristics of included studies

#### Study design

This review includes *n* = 14 RCTs [20–33], *n* = 2 CTs [34, 35], and *n* = 3 UCTs [36–38].

#### Cohort characteristics

The total number of participants was *N* = 780, of whom *n* = 760 completed the interventions. Sample sizes of the single studies ranged from *n* = 10 to 242 participants. Three studies included female pwMS only [20, 24, 34]. All other studies had both female and male participants. Mean age of participants ranged from 28.3 [21] to 60.1 [33] years. Mean time since diagnosis ranged from 6.2 [22] to 22.3 [26] years. Mean disease severity (Expanded Disability Status Scale (EDSS) score) ranged from 1.5 [36] to 6.1 [29]. Considering the MS phenotypes, *n* = 6 studies included participants with relapsing–remitting MS (RRMS) phenotype only [20, 21, 24, 30, 31, 34]. A single study investigated participants with secondary progressive MS (SPMS) only [36]. Eight studies included all three phenotypes (RRMS, SPMS, PPMS) [23, 25, 27, 32, 33, 35, 37, 38] and one study included participants with the RRMS and SPMS phenotypes [26]. Three studies did not provide any information regarding the MS phenotypes of participants [22, 28, 29].

#### Interventions

#### Frequency

Participants (initially) trained two [22–24, 26, 27, 29, 32, 38] or three times per week [20, 21, 25, 28, 30, 31, 33–37]. In the majority of studies (*n* = 12), the training frequency was three times per week. The intensity was progressively increased in almost all studies with the exception of two studies [36, 38].

#### Intensity

Studies employed different measures to prescribe exercise intensity of functional exercise training. Most often, measures of internal load, which refer to the psychophysiological responses that occur during the exercise (e.g., Borg’s rate of perceived exertion (RPE) or Category Ratio (CR)−10 scale scores), were combined with measures of external load, which refer to the physical exertion during the exercise (e.g., exercise volume (i.e., repetitions x number of sets)) [39, 40]. Prescriptions of internal load were most frequently based on self-report measures (*n* = 6), including the RPE scale [20, 26, 27, 37] and the CR-10 scale [30, 32], percentage of the heart rate maximum (% HR_max_) [27], or % of maximal tolerated power [22]. Four studies allowed participants to self-regulate internal load [23, 24, 29, 36]. Information on internal load was absent in *n* = 7 studies [21, 25, 28, 31, 33–35], and partly missing in two other studies [22, 38]. Prescription of external load was based on exercise volume (i.e., repetitions x number of sets) in most studies (*n* = 15) [20, 21, 23–28, 30–35, 38]. Some studies used measures such as V̇O_2peak_ [21], HR_max_ [20, 27], HRR [24], the (modified) RPE scale [20, 27, 30, 32, 37], a percentage of the body weight [25, 28], the maximal tolerated power [38] or the workload [22], while others controlled intensity through the number of sets and repetitions of exercises [36, 38] or allowed participants to self-regulate [23, 24, 27, 29]. Participants performed between one to five sets of five to 15 repetitions each. Duration of training sessions ranged between 30 and 120 min.

#### Time

Interventions lasted six [23, 36], eight [20–22, 25, 28, 30–32, 34, 37], ten [27], twelve [5, 24, 26, 32, 38], or 16 [35] weeks. The duration of most studies was eight weeks (*n* = 10).

#### Type

Target muscle groups of training programs differed. Nine studies conducted a whole body resistance training, including the muscle groups of the upper and lower lips, as well as the trunk [20, 21, 24, 27, 29, 30, 32, 34, 35], or a training program solely including lower body resistance training exercises (*n* = 7) [22, 23, 25, 26, 28, 37, 38]. Two studies primarily focused on resistance training of the trunk muscle [31, 36]. Additionally, seven studies included forms of endurance training and strength training in their interventions [5, 20, 21, 24, 27, 37, 38]. In six studies, balance exercises were also incorporated as part of the training [22, 26, 29, 31, 33, 37]. A single study performed lower body plyometric exercise, combined with balance exercise [22], and one study combined for hip and core [33]. Exercises were performed with body weight only [31] or with additional equipment. Additional equipment included elastic bands [20, 21, 24, 26, 30, 32, 33, 35, 37, 38], suspension trainers (TRX^®^) [20, 34], weighted vests [21, 25, 35], ankle weights [23, 25, 32, 38], dumbbells [23, 24, 29], a weighted backpack [23], barbells [23–25, 27–29, 32, 35, 38], medicine balls [36], or free weights not specified in detail [27, 28].

Table 2 shows the interventions, summarized according to the FITT (frequency, intensity, time, type) criteria. See supplementary material for a more detailed table (Table 3).

**Table 2:** Functional exercise training studies in persons with multiple sclerosis

	Design	Sample Size	Age [yrs]	EDSS	MS type	Frequency	Intensity	Time	Type	Outcomes	Between-group
Abbaspoor et al. 2020	RCT	INT: 10	33.50 ± 6.37	3.06 ± 1.20	RRMS	3x/week 8 weeks	RPE 10–16 55–70% HR_max_	5 exercises; 1–2 sets × 8–14 reps; 60–120 s rest 15–20 min	*WB resistance* BW; elastic bands; TRX® suspension *Rhythmic AT*	Serum markers (BDNF, IGF-1); walking speed (10MWT); walking endurance (2MWT); quadriceps strength (dynamometry); handgrip/finger strength (dynamometry)	INT: IGF-1 ↑; 10MWT ↑; Handgrip strength (R) ↑; BDNF ↔; 2MWT ↔; Handgrip strength (L) ↔; Finger strength (R/L) ↔
		CON: 10	36.75 ± 6.80	3.00 ± 1.10					Habitual lifestyle		
Akbar et al. 2020	CT	INT1: 5	45.6 ± 12.8	N/A	RRMS SPMS PPMS	3x/week 16 weeks	N/A	10 exercises; 1–3 sets × 10–15 reps	*WB resistance* BW; elastic bands; weighted vests *Stretching*	Fatigue (Modified Fatigue Impact Scale (total, physical, cognitive, psychosocial); physical activity (Godin Leisure-Time Exercise Questionnaire); grip strength (dynamometry); brain connectivity (resting-state functional MRI)	INT1 vs. INT2: Functional connectivity caudate and inferior parietal region (L), inferior frontal region (R/L), middle frontal region (L), insula (R) ↕; modified Fatigue Impact Scale (total, physical, cognitive, psychosocial) ↔; Godin Leisure-Time Exercise Questionnaire ↔; grip strength
		INT2: 5	53.0 ± 10.7						INT2: Stretching		
Ayán Pérez et al. 2007	UCT	INT: 24 (36)^1^	44.4 ± 9.5	1.5 ± N/A	SPMS	3x/week 6 weeks	Self-chosen (2–3 reps less than subjective max)	12 exercises; 1–2 sets x individual number of reps; ~ 2 min medicine ball throws; 60 min	*Core strength and WB mobility* BW; Calisthenics; medicine balls	Walking speed (9 m zig-zag run); mobility of arms and time–space orientation (clapping test, dynamic flexibility test); explosiveness of arms (overhead medicine ball throw); explosiveness of legs (vertical jump); trunk strength (abdominal test, back muscle test) leg lifts, Kraus-Weber test); Balance (flamingo balance test)	-
Bilek et al. 2022	RCT	INT: 30	28.29 ± 6.57	1.71 ± 1.13	RRMS	3x/week 8 weeks	N/A 70% V̇O_2peak_	60–70 min; 10 exercises; 1–3 sets × 10–15 reps; rest N/A 30 min	*WB resistance* Elastic bands; weighted vests *AT (bicycle)*	Cardioresp. fitness (V̇O_2 peak_); serum markers (Contactin-1, Contaction-2); Cognition (PASAT-3)	INT vs. CON: V̇O_2 peak_ ↑; Contactin-1 ↑; Contaction-2 ↑; PASAT-3 ↑
		CON: 30	31.36 ± 8.07	2.00 ± 1.14					Habitual lifestyle		
Cakt et al. 2010	RCT	INT1: 10	43.0 ± 10.2	N/A	RRMS SPMS	2x/week 8 weeks	N/A	15–25 min LB plyometrics + balance; 2 exercises LB plyometrics 15 sets × 2 min high-resistance cycling, 2 min low-resistance/rest	*INT1: LB plyometrics* * INT 1: Balance exercise* Walking, standing, balance board	Mobility (TUG); dynamic balance (Dynamic Gait Index); static balance (Functional Reach); fear of falling (Falls Efficacy Scale); walking speed (10MWT); fatigue (Fatigue Severity Scale); depression (Beck Depression Inventory); HRQoL (Short Form-36); total maximum workload; exercise duration	INT2 vs. INT 1 vs. CON: TUG ↑; Dynamic Gait Index ↑; Functional Reach ↑; Falls Efficacy Scale ↑; Fatigue Severity Scale ↑; Beck Depression Inventory ↑; total maximum workload ↑; exercise duration ↑; 10MWT ↔; SF-36 ↔ INT2 vs. CON: 10MWT ↑
		INT2: 14	36.4 ± 10.5				40% total maximum workload		INT 2: Additional* High-resistance cycling* Bicycle ergometer		
		CON: 9	35.5 ± 10.9						Habitual lifestyle		
Coote et al. 2015	RCT	INT1: 10	51.8 ± 12.1	N/A	RRMS SPMS PPMS BMS	2x/week 6 weeks; 3x/week 6 weeks	Adj. until muscular failure	6 exercises; 1–3 sets × 12 reps, 2–3 min rest	*INT1: LB resistance* BW, wooden box, free weights (hand, ankle, backpack)	Knee extension strength (dynamometry); hip extension strength (dynamometry); lower extremity strength (maximum rep Sit To Stand Test); lower-limb spasticity (VAS); mobility (TUG); self-reported walking impairment (MS Walking Scale–12); balance (Berg Balance Scale); impact of MS (MS Impact Scale-29); fatigue (Modified Fatigue Impact Scale)	INT1 vs. INT2: Modified Fatigue Impact Scale (total) ↓; hip/knee extension strength ↔; Sit To Stand ↔; spasticity VAS ↔; TUG ↔; MS Walking Scale-12 ↔; MS Impact Scale (physiological/psychological score) ↔; Berg Balance Scale ↔
		INT2: 15	51.8 ± 12.6						*INT2: LB resistance combined with neuromuscular electric stimulation* s.a		
Correale et al. 2021	RCT	INT: 14	45.4 ± 7.2	2.25 ± 0.8	RRMS	2x/week 12 weeks	Self-chosen	45–60 min; 3 sets × 8–12 reps; 60–90 s rest 25 min; 50–70% HRR	*WB resistance* Calisthenics; dumbbells; elastic bands *AT *(bicycle/treadmill)	Strength quadriceps (MVIC, 1-RM leg extension); strength chest (1-RM chest press); strength back (1-RM seated row); fatigue (MFIS); depressive symptoms (BDI-II); HRQoL (MSQOL-54)	INT vs. CON: MFIS ↑; MSQOL-54 (mental composite) ↑; MVIC ↔; 1-RM leg extension ↔; 1-RM chest press ↔; 1-RM seated row ↔; depressive symptoms ↔; MSQOL-54 (physical composite) ↔
		CON: 13	48.3 ± 6.1						Habitual lifestyle		
De Bolt and McCubbin 2004	RCT	INT: 19	51.63 ± 6.71	3.97 ± 1.79	RRMS PMS BMS	3x/week 8 weeks	≥ 0.5% BW	25–30 min, 2–3 sets × 8–12 reps; self-chosen rests	*LB resistance* BW, weighted vests, ankle weights, step	Balance (anteroposterior sway, mediolateral sway, sway velocity); leg extensor power (maximal leg extension power); mobility (TUG)	INT vs. CON: absolute/relative leg extensor power ↑; anteroposterior/mediolateral sway ↔; sway velocity ↔; TUG ↔
		CON: 18	47.78 ± 10.47	3.50 ± 1.50					Habitual lifestyle		
Frevel and Mäurer 2015	RCT	INT1: 9	44.3 ± 8.1	3.8 ± 1.5	RRMS 6 (67) SPMS 3 (33)	2x/week 12 weeks	RPE 11–14	45 min; 5–8 exercises; 2–3 sets × 8–15 reps,	*INT1: LB resistance* BW, elastic bands, gym ball, mat * INT1: Balance* Standing, unstable surface	Balance (Berg Balance Scale; Dynamic Gait Index); knee extensor/flexor strength (MVIC); trunk extensor/flexor strength (MVIC); mobility (TUG); walking speed (2MWT 1 min/total); HRQoL (Hamburg Quality of Life Questionnaire in Multiple Sclerosis (HAQUAMS, total, all subscales); fatigue (Fatigue Severity Scale; Modified Fatigue Impact Scale)	INT vs. INT2: MFIS cognitive subscale ↑; HAQUAMS upper limb ↑; Berg Balance Scale ↔; Dynamic Gait Index ↔; knee extensor/flexor MVIC ↔; trunk extensor/flexor MVIC ↔
		INT2: 9	46.9 ± 7.6	3.8 ± 1.1				20–30 min	INT2: Hippotherapy		
Garrett et al. 2013	RCT	INT1: 63^#^	51.7 ± 10	N/A	RRMS SPMS PPMS BMS	1x/week 10 weeks 2x/week 10 weeks	Until muscular failure 65% HR_max_, RPE 11–14	60 min; 3 sets × 12 reps 30 min 30 min	*INT1: WB resistance* BW, free weights * INT1: AT* Mode of choice (walking/cycling/swimming/running)	HRQoL (Multiple Sclerosis Impact Scale-29 (physical/psychological)); fatigue (Modified Fatigue Impact Scale (total/physical/cognitive)); walking endurance (6MWT)	INT1 vs. CON: Multiple Sclerosis Impact Scale-29 (physical/psychological) ↑; Modified Fatigue Impact Scale (total/physical) ↑; Modified Fatigue Impact Scale (cognitive) ↔; 6MWT ↑ INT2 vs. CON: Multiple Sclerosis Impact Scale-29 (physical/psychological) ↑; Modified Fatigue Impact Scale (total/physical) ↑; Modified Fatigue Impact Scale (cognitive) ↔; 6MWT ↑ INT3 vs. CON: Multiple Sclerosis Impact Scale-29 (psychological) ↑; Modified Fatigue Impact Scale (total/physical) ↑; Multiple Sclerosis Impact Scale-29 (physical) ↔; Modified Fatigue Impact Scale (cognitive) ↔; 6MWT ↔
		INT2: 67^#^	50.3 ± 10			1x/week 10 weeks	Not predefined	Not predefined	*INT2: Resistance training/AT/combined training* Not predefined		
		INT3: 63^#^	49.6 ± 10			1x/week 10	Not predefined	Not predefined	*INT3: Yoga* Not predefined		
		CON: 49^#^	48.8 ± 11						Habitual lifestyle		
Hosseini et al. 2018	RCT	INT1: 9	32.9 ± 8.1	N/A	N/A	3x/week 8 weeks	≥ 1% bodyweight	5 exercises; 3 sets × 10 reps, 30–60 s rest (betw. exercises)	*INT1: LB resistance* Free weights (fastened to the body)	Leg extensor strength (1-RM); leg strength (leg press); walking speed (10MWT); balance (postural sway balance index, two legs open/closed eyes, one leg open eyes)	INT1 vs. INT2 vs. CON: Leg extensor 1-RM ↕ (INT1 ↑)
		INT2: 9	31.3 ± 7.1					60–70 min; 1 set × 30–60 s, 30–60 s rest	*INT2: Hatha Yoga* Standing, sitting, lying		
		CON: 8	33.0 ± 9.7						Habitual lifestyle		
Learmonth et al. 2012	RCT	INT: 20	51.4 ± 8.06	6.14 ± 0.36	N/A	2x/week 12 weeks	Self-chosen	30–40 min; 8–12 exercises; N/A sets × 1 min; ≥ 60 s rest	*WB resistance* BW, dumbbells, chair *AT* Walking/stepping/bike/Foot pedals *Balance* Sitting/standing/walking	Walking speed (T25FW); body mass index; walking endurance (6MWT); balance (Berg Balance Scale); mobility (TUG); quadriceps strength (maximum isometric force leg extension, weaker leg); activity level (PhoneFITT); balance confidence (Activities Balance Confidence); fatigue (Fatigue Severity Scale); anxiety and depressive symptoms (HADS); HRQoL (LMSQOL); goal attainment (Goal Attainment Scale)	T25FW ↔; PhoneFITT ↕; Activities Balance Confidence ↑ (0–12 wks); body mass index ↔; 6MWT ↔; Berg Balance Scale ↔; TUG ↔; maximum isometric force leg extension ↔; fatigue severity scale ↔; HADS ↔; LMSQOL ↔
		CON: 12	51.8 ± 8.0	5.82 ± 0.51					Habitual lifestyle		
Mardaniyan Ghahfarrokhi et al. 2022	RCT	INT1: 15	39.87 ± 9.09	4.13 ± 0.97	RRMS	3x/week 8 weeks	CR-10: 2–6	120–190 min, 14 exercises, 1–2 sets × 10–12 reps	*INT1: WB resistance* Elastic bands	Static balance (wide/narrow, single-leg (R/L, open/closed eyes), semi-tandem, tandem open/closed eyes); dynamic balance (tandem walk test); mobility (TUG); walking endurance (6MWT); walking speed (10MWT, T25FWT); walking ability (6 Spot Step Test (dominant/non-dominant); lower extremity strength (5 Times Sit to Stand); hand grip strength (dynamometry)	INT1 vs. INT2: Tandem walk ↓; Six Spot Step Test (non-dominant) ↓; 6MWT ↓; all other outcomes ↔
		INT2: 15	37.50 ± 8.58	4.57 ± 1.30			CR-10: 2–6	90–120 min, 2 sets × 8 reps, 2 min rest	*INT2: Neurofunctional exercise* Balance (standing, walking), walking, WB resistance, pelvic control, core stability, BW, cones, Swiss ball		
Moghadasi et al. 2020	CT	INT: 19	37.62 ± 4.58	2.18 ± 0.85	RRMS:	3x/week 8 weeks	N/A	30 min; 8 exercises; 3 sets × 5–10 reps	*WB Resistance* TRX® suspension	Mobility (TUG); walking speed (10MWT); walking endurance (2MWT); lower extremity strength (5 Times Sit to Stand); joint position sense (knee proprioception absolute error); quadriceps/knee extensor strength (MVIC); knee flexor strength (MVIC)	INT vs. CON: TUG ↑; 10MWT ↑; 2MWT ↑; 5 Times Sit to Stand ↑; knee proprioception absolute error non-dominant leg (60°) ↑;MVIC knee extensors dominant/non-dominant (20° and 70°) ↑; MVIC knee flexors dominant/non-dominant (20°) ↑; MVIC knee flexors dominant (70°) ↑; knee proprioception absolute error dominant leg (60°) ↔; proprioception absolute error dominant and non-dominant leg (30°) ↔; MVIC knee flexors non-dominant (70°) ↔
		CON: 15	34.72 ± 5.01	2.59 ± 1.01					Habitual lifestyle		
Motl et al. 2012	UCT	INT: 13	51.5 ± 11.3	5.58 ± 0.79	RRMS SPMS PPMS	3x/week 8 weeks	RPE 13 RPE 13 RPE 13	15–60 min; 5–20 min; 5 exercises; sets/reps N/A 5–20 min 5–20 min	*LB resistance* Elastic bands *AT* Ergometry (bicycle, rowing, elliptical)/treadmill *Balance* Standing, walking	Walking speed (T25FW); self-reported walking impairment (MS Walking Scale-12); mobility (TUG); gait efficiency (Functional Gait Profile score, spatial and temporal gait measures)	–
Najafi et al., 2019	RCT	INT: 30	38.39 ± 4.59	2.51 ± 1.22	RRMS	3x/week 8 weeks	N/A	45–60 min; 5 exercises; 3–4 sets × 10–15 s, 10–15 s rest	*Core training* BW *Balance* Standing, walking (open/closed eyes)	Postural control (Center of Pressure sway area and path length, opened/closed eyes); balance (Berg Balance Scale); walking speed (T25FW); mobility (TUG)	Center of Pressure sway area (opened/closed eyes) ↑; Center of Pressure path length (opened/closed eyes) ↑; Berg Balance Scale ↑; T25FW ↑; TUG ↑
		CON: 30	36.36 ± 3.54	2.44 ± 0.77					N/A		
Sabapathy et al. 2011	RCT	INT1: 11 (14)^1^	55 ± 7	N/A	RRMS SPMS PPMS	2x/week 8 weeks	CR10 3–5	8 exercises, 2–3 sets × 6–10 reps, ≥ 30–60 s rest 15–20 min	*INT1: WB resistance and balance* Elastic band, dumbbell (1–4 kg), parallel bar, Swiss ball, ankle weights (1–5 kg), foam mat, foam beam, wobble board *INT1: Stretching*	Grip strength (dynamometry); static balance (functional reach), dynamic balance (4 Step Square); mobility (TUG); walking endurance (6MWT); HRQoL (Multiple Sclerosis Impact Scale (physical/psychological); Short Form-36 (Physical and Mental Component Summary); fatigue (Modified Fatigue Impact Scale (physical/cognitive/psychosocial)); depressive symptoms (Becks Depression Inventory)	INT1 vs. INT 2: all outcomes ↔
		INT2: 5 (6)^1^						8 stations × 5 min, 2 min rest every 10 min 15–20 min	*INT2: AT* Arm crank, cycling, cross-trainer, step-ups, recumbent cycling, treadmill INT2: Stretching		
Sosnoff et al. 2014	RCT	INT: 13	60.1 ± 6.3	5.5 (2.5)	RRMS SPMS PPMS	3x/week 12 weeks	N/A	45–60 min; 3 sets × 8–12 reps	*LB and core resistance* Elastic bands, BW *Balance* Standing, walking	Fall risk (Physiological Profile Assessment total, subcomponents); balance (Berg Balance Scale); balance confidence (Activities-Specific Balance Confidence scale); walking speed (T25FW); walking endurance (6MWT); mobility (TUG); self-reported walking impairment (MS Walking Scale-12); self-reported fall frequency	INC vs. CON: Physiological Profile Assessment total ↑; postural sway subcomponent ↑; T25FW ↑; Activities- Specific Balance Confidence scale ↑; other subcomponents (Melbourne Edge detection test, proprioception, strength, reaction time) ↔; 6MWT ↔; TUG ↔; MS Walking Scale-12 ↔; Berg Balance Scale ↔
		CON: 14	60.1 ± 6.0	5.5 (3.5) [Mdn (IQR)]					Habitual lifestyle		
Zaenker et al. 2018	UCT	INT: 26 (30)^1^	44.6 ± 7.9	2.46 ± 1.52	RRMS SPMS PPMS	2x/week 1–4 week 3x/week 5–12 week	N/A Intervals at 90–110% maximal tolerated power, rest at anaerobic threshold moderate	4 exercises, 4–5 sets × 10–15 reps s.a 17 min 35–45 min	*LB resistance* BW, resistance band, ankle weight *Additional LB resistance* (5–12 weeks, every 2nd week) *AT* HIIT: 5 intervals, 3 min rest *Additional AT *(5–12 weeks, every 2nd week) Self-chosen	Cardiorespiratory fitness (VO_2 peak_, lactate at the end of the maximum aerobic test, HR_peak_), physical capacity (maximal tolerated power), quadriceps strength (isokinetic peak torque), hamstring strength (isokinetic peak torque); HRQoL (MSQOL-54 total, subscales)	–

Intervention duration without warm-up/cool-down periods; ^#^whole group prior drop-out; bold = in multicomponent interventions considered as “functional training”

↔  = no change, ↑ = significant improvement/between-group effect in favor of INT(1); ↓ = significant worsening/between-group in favor of INT2/CON; ↕ = unspecified significant difference, ^1^Data presented for n completing the program, initial sample size in brackets

*AT* aerobic training, *BDNF* brain-derived neurotrophic factor, *BDI-II* Beck depression inventory-II, *BMS* benign MS, *CPET* cardiopulmonary exercise test, *CON* control group, *CT* controlled trial, *EDSS* expanded disability status scale, *f* female, *HRR* heart rate reserve, *HADS* hospital anxiety and depression scale, *HAQUAMS* Hamburg quality of life questionnaire in multiple sclerosis, *HR*_*max*_*/HR*_*peak*_ maximum/peak heart rate, *HRQoL* health-related quality of life, *IGF-1* insulin like growth factor, *INT* intervention group, *L* left, *LB* lower body, *m* male, *LMSQOL* Leeds multiple sclerosis quality of life scale, *MFIS* modified fatigue impact scale, *MRI* magnetic resonance imaging, *MSQOL-54* multiple sclerosis quality of life instrument-54 item, *MVIC* maximal voluntary isometric contraction, *PASAT-3* paced auditory serial addition test with 3 s stimulus, *PMS* progressive MS, *PPMS* primary progressive MS, *PSFS* patient-specific functional scale, R right, *RCT* randomized controlled trial, *RPE* rate of perceived exertion, *RRMS* relapsing–remitting MS, *RT* resistance training, *SF-36* Short Form-36, *SPMS* secondary progressive MS, *TUG* timed up and go, *TSD* time since diagnosis, *T25FW* timed 25-foot walk, *UCT* uncontrolled trial, *VAS* visual analog scale, V̇*O*_*2 max*_*/V̇O*_*2 peak*_ maximum/peak oxygen consumption, *WB* whole body, *1-RM* 1-repition maximum, *2MWT* 2-min walk test, *6MWT* 6-min walk test, *10MWT* 10-m walk test

#### Control group

Three out of the 21 included studies had no control group [36–38]. Among the remaining studies, two did not provide information on how the control condition was designed [24, 31], and nine instructed the control group to continue their usual routines and activities [20–22, 25, 27–29, 33, 34].

#### Assessment tools

The included studies used a variety of different assessments to quantify mobility (Timed Up and Go Test (TUG)) [22, 23, 25, 26, 29–34, 37], walking performance on short walking tests (e.g., Timed 25-Foot Walk (T25FW), Ten-Meter Walk Test) [20, 22, 28–31, 33, 34, 36, 37], walking performance on long walking tests (e.g., Two-/Six-Minute Walk Test (6MWT)) [20, 26, 27, 29, 30, 32–34], strength (e.g., one repetition maximum (1RM), maximum voluntary isometric contraction (MVIC)) [20, 23–26, 28–30, 32, 34–36, 38], and cardiorespiratory fitness (e.g., V̇O_2peak_, HR_max_, or blood pressure) [21, 38]. Furthermore, heterogeneous tests were used to assess static and dynamic balance and postural control (e.g., Berg Balance Scale, Flamingo Balance Test, Functional Reach Test, postural sway) [22, 23, 25, 26, 28–34, 36]. Patient-reported outcomes assessed in the included studies were health-related quality of life (Leeds Multiple Sclerosis Quality of Life scale (LMSQOL), Multiple Sclerosis Quality of Life (MSQOL)), fatigue (i.e., Modified Fatigue Impact Scale (MFIS) and/or the Fatigue Severity Scale) [22, 24, 26, 27, 29, 32, 35], and mood (e.g., Beck Depression Inventory-II (BDI-II) or Hospital Anxiety and Depression Scale (HADS)) [22–24, 26, 27, 29, 32, 38]. Physiological measures were infrequently assessed and included blood-derived markers (e.g., serum contaction-1/−2 BDNF, IGF) and brain connectivity metrics (i.e., Akbar et al. [35]). Individual studies investigated effects of functional exercise on self-reported walking impairment or balance confidence, body composition (body mass index), cognition, spasticity, spatial and temporal gait kinematics, fall risk, coordination, flexibility, and change in physical activity behavior [20, 21, 23, 29, 33, 35, 37].

### Study quality, reporting, and risk of bias

The average study quality was 8.63 points, with twelve studies scoring ≥ 8 points [5, 20–27, 30, 32, 33], five studies falling within the range of 4 to 7 [28, 31, 34, 35, 37], and two falling ≤ 3 [36, 38]. None of the 19 studies fulfilled all items on the 15-point scale. Study quality was on average 3.05 points (range 0–5) out of five possible points and study reporting was on average 5.58 points (range 2–9) out of a possible 10 points. Attendance of the participants was reported in 11 of the 19 studies, and within the TESTEX evaluation, 52.6% of the studies achieved more than 85% [21–33, 36–38]. No specific information on exercise training adherence and whether the participants performed the training sessions in line to the prescribed modalities (e.g., duration, type, intensity, frequency) was provided. In all studies reporting adverse events (*n* = 5), no (serious) adverse events occurred [22, 23, 30, 32, 33]. Only two studies conducted an intention-to-treat analysis [5, 30], while the other studies did not perform this analysis or did not describe their statistical approach more explicitly. Table 1 shows the results of the TESTEX quality assessment. Overall risk of bias was high or of some concerns in the majority of included studies, mainly due to concerns in reporting (domain 5) and deviations from the intended interventions (domain 2). Measurement of the outcome was at low risk in the most studies (domain 4). An overview of the risk of bias assessment is described in supplementary material 2 (risk of bias of the included studies).

### Effects of functional exercise training

#### Between group

A total of sixteen studies reported significant between-group effects in the assessed outcomes [5, 20–28, 30–35]. In the studies that examined mobility, significant improvements were recorded in three studies in favor of the intervention group compared to the control group [22, 31, 34] and seven studies did not observed significant differences between the groups [23, 25, 26, 29, 30, 32, 33]. Walking performance on short walking tests was significantly increased in four studies compared to the control group [20, 31, 33, 34], while no differences between the groups were found in four studies [22, 28–30]. Walking performance on long walking tests were significantly increased the intervention group compared to the control group in two studies [27, 34], while five studies found no significant differences between the groups [20, 26, 29, 32, 33], and in the intervention of Mardaniyan Ghahfarrokhi et al. [30], the result of the intervention group was significantly decreased compared to the control group. Regarding strength, significant increases were found in four studies in the intervention group compared to the control group [20, 25, 28, 34], no significant changes were reported in six studies [24, 26, 29, 30, 32, 35], while in Coote et al.’s study [23] the intervention group showed significant decrease in contrast to the comparison group. Furthermore, V̇O_2peak_ examined by Bilek et al. [21] significantly increased in the intervention group compared to the control group. Within the studies that examined balance, results of four studies showed significant improvements in the intervention group compared to the control group [22, 29, 31, 33], while nine studies found no significant changes [23, 25, 26, 28–30, 32–34]. In two studies, the Balance Confidence Scale was significantly improved compared to the control group, while at the same time no changes were achieved in the Berg Balance Scale [29, 33]. Regarding fatigue, four studies achieved significantly improved scores in at least one subcategory compared to the control group [22, 24, 26, 27] and three studies found no significant change in fatigue scores [29, 32, 35]. Additionally, concerning quality of life and depression significant improvements in at least one subcategory compared to the control group were observed in four studies [22, 24, 26, 27], while three studies found no significant differences compared to the control group [23, 29, 32]. Three studies investigated the effects of the training on blood-based measures. Bilek et al. [21] found significant increases in contactin-1 and −2 concentration compared to the control group, Abbaspoor et al. [20] observed significant improvements for IGF. However, the BDNF concentration did not show significant changes in comparison to the control group [20]. Akbar et al. [35] showed significant differences in brain connectivity compared to the control group.

#### Pre–post-test

A total of three studies documented pre–post-test outcomes [36–38], which are presented in Table 2.

## Discussion

This systematic review aimed to summarize the current evidence on the effects of functional exercise training in adult pwMS. Our literature search yielded 19 eligible articles, that revealed large heterogeneity in terms of intervention design, methodological quality, and outcomes chosen.

### Interventions

The heterogeneity of intervention design is not surprising, given the absence of a unified taxonomy of functional training but, instead, a large number of parallel definitions and interpretations [41]. In this review, we operationalized functional training as training programs encompassing exercises that are performed with body weight and/or the use of additional small equipment, and, thus, are not reliant on strength training machines [17]. Accordingly, most studies used a variety of small equipment, such as elastic bands, weighted vests, ankle weights, dumbbells, or suspension trainer systems (TRX^®^) to feature body weight exercises, such as squats, lunges, and other lower-extremity exercises. Although the strength training protocols used in the included studies were generally well designed and largely consistent, the choice and number of exercises may have had a greater impact on outcomes. Variations in exercise selection and volume could influence the effectiveness of the interventions, potentially contributing to differences in strength improvements across studies.

Unfortunately, many studies did not or only partially describe the exercises and exercise designs in detail. This aspect may be particularly critical, when considering safe exercise execution and potential ways of adaption or support for pwMS with advanced disability in clinical practice. However, there were some studies providing definitions or examples exercises [21, 22, 24, 26–28, 33, 35, 37] or detailed lists of exercise [20, 23, 25, 29–32, 34, 36, 38]. Within these lists, intended exercises per week along were provided along with details on exercise execution and required materials, which is essential to create personalized and tailored exercise programs for individual pwMS.

### Study quality, reporting, and risk of bias

According to the TESTEX tool, average study quality of the included studies was 8.63 points out of 15 possible points (57.54%) with a range of two to 14 points, indicating a very broad spectrum of methodological quality across the studies and an overall moderate study quality. This is also reflected in the risk of bias assessment, which indicates a high level of risk or some concerns in the overall evaluation. Some aspects, such as the reporting of results and the absence of intention-to-treat analysis or allocation concealment, are reflected in both the TESTEX tool criteria and the RoB 2 tool subdomains. The RoB-2 assessment indicates that the measurement of outcomes was methodologically well implemented in most of the studies included in this review, resulting in a low risk of measurement bias. However, methodological weaknesses were observed in how dropouts and missing data were handled, increasing the risk of bias due to incomplete outcome data (attrition bias). This was particularly evident when there were no plausible explanations or adequate statistical procedures to account for the dropouts. Furthermore, the reporting on attendance appears to be rather low across the included studies and reasons for not attending training sessions were generally poorly reported. Various personal and disease-related events (e. g., illness, specific MS-related symptoms, holidays etc.) may occur that prevent a consistent completion, attendance, and adherence to exercise sessions. This issue has also been observed in other exercise trials in pwMS, potentially leading to heterogeneous results in outcome measures [42]. By clearly reporting these parameters and considering reasons for reduced exercise adherence and attendance in future studies, more reliable conclusions can be drawn about individual factors, especially given the diversity in how pwMS respond to exercise interventions. Although afflicted by poor reporting in most studies, functional exercise appears not to be associated with adverse events in persons with no, mild, and moderate disability (≤ EDSS 6.0) [22, 23, 30, 32, 33]. Therefore, it can be concluded that people with an EDSS ≤ 6 can engage in functional training. This adaptability is due to the fact that functional training can be tailored to individual differences through the variability of exercises and the minimal need for equipment.

### Effects of functional exercise training

In this review, we did not perform any restriction to specific outcomes, resulting in a diverse set of outcomes assessed in individual studies. A large number of these heterogeneous outcomes improved after performing functional training in both controlled and uncontrolled studies.

#### Effects of functional exercise training on mobility and walking capacity

Mobility limitations and gait issues are among the most common symptoms or impairments in pwMS [43, 44]. The heterogeneous results may be due to a number of factors. For instance, the choice of measurement methods is different and varies in terms of mobility and walking performance. Another reason could be the different training protocols of the interventions. All the trials that showed improvements in all their outcomes measures included mildly affected participants (mean EDSS < 2.5) [31, 34], while the studies that showed no improvement or only partial improvement included participants with more progressed disability (mean EDSS 4.6) [20, 25, 29, 33]. PwMS having a EDSS score < 4.5 are usually fully ambulatory and may therefore respond better to functional training programs, supporting the theory of a “window of opportunity” for exercise therapy in pwMS starting early after diagnosis [45]. As the disease progresses and EDSS scores increase, mobility decreases, which may affect adaptability [46, 47]. The progression of the disease and the resulting worsening of physical and mental capacity can limit functional training. However, a major advantage of functional training compared to other training modalities (e.g., isolated endurance or strength exercise) is that it offers a wide range of adjustment options. For instance, more complex movement sequences that are no longer comprehensible due to deteriorating cognition can be replaced with simpler exercises. Training durations can also be shortened as fatigue worsens, and exercises can be performed while sitting down instead of standing up, given that many people with MS experience worsening balance issues as the disease progresses.

#### Effects of functional exercise training on strength measures

Examining the results concerning strength, testing methods and results varied considerably. Significant increases in muscle strength after the intervention can be seen in the majority of the studies, indicating a general improvement in strength performance in at least one of the measured strength measures when comparing the pre-test and post-test results [20, 22–25, 28, 30, 34–36, 38]. However, in one study, no improvement in knee and trunk flexor and extensor strength was noted [26]. The previously tested muscles are predominantly trained by the single exercises and it is therefore an expected result that the muscles improve after regular training over a period of time. Although the mechanisms behind muscle loss in individuals with disabilities and its resulting impact on reduced mobility and functional limitations remain not fully understood [48], research indicates that resistance training can play a crucial role in preserving or enhancing muscle strength [49–51]. This is further corroborated by the studies included in this review.

#### Effects of functional exercise training on cardiovascular measures

Although seven studies included endurance or aerobic training in their interventions [5, 20, 21, 24, 27, 37, 38], only two studies assessed changes in cardiovascular fitness (i.e. V̇O_2 peak_).[21, 38]. Among those, V̇O_2peak_ was improved in both studies [21, 38]. Aerobic capacity is considered to be an important physiological measure in pwMS, which is associated with exercise-induced improvements and subsequent health benefits [52]. Therefore, it could be useful to include cardiovascular measures in future studies, even if functional exercise training does not consist solely of endurance training elements.

#### Effects of functional exercise training on balance and proprioception

The studies investigated balance using different assessments, yielding heterogeneous results. Interestingly, in two studies, the Balance Confidence Scale was significantly improved while at the same time no changes were achieved in the Berg Balance Scale [29, 33]. This indicates that the participants subjectively rated their balance ability as improved and the intervention may have increased their confidence in dealing with balance situations, while objectively no improvement could be measured. This result confirms the assumption that physical exercise can improve self-efficacy in pwMS, which has already been shown in studies [53, 54].

#### Effects of functional exercise training on patient-related outcomes measures (PROMS)

Only seven out of 21 studies investigated possible changes using specific fatigue questionnaires (Fatigue Impact Scale, Fatigue Severity Scale), although fatigue being a typical symptom of MS. In four out of seven studies that assessed quality of life, no changes were found after the intervention compared to the control group [22, 23, 29, 32], while two studies achieved improvements [26, 27]. The subjects in the intervention of Correale et al. [24] reached improvements in the mental composite but not in the physical composite. The results suggest that functional exercise training may not be the right intervention to increase QoL, here large randomized controlled trials could provide more definitive evidence. A notable limitation in the interpretation of PROMS outcomes is that many included studies primarily investigated participants with mild MS symptoms. Consequently, if only a small proportion of participants experience, for instance, fatigue or reduced quality of life at baseline, the potential for measurable improvement due to an intervention is inherently limited. Additionally, several of the applied measurement tools may not have been sensitive enough to detect meaningful changes, further complicating the assessment of intervention effects.

#### Effects of functional exercise training on blood-based measures

Significant improved results were observed concerning contactin 1, contactin 2, and IGF [20, 21]. Contrary to expectations, no changes in BDNF levels were evident. In other reviews examining the impact of physical activity on BDNF, significant increases in BDNF levels were at least partly observed. However, these studies varied greatly in their intervention modalities, making it difficult to draw specific conclusions about why BDNF levels increased in some cases and not in others [55, 56]. Future research should consider blood-based measures in their interventions, as the evidence for the effects of physical training on markers of neuronal damage and markers of disease activity in MS patients is very limited [57], but animal studies particularly indicate a disease-modulating effect of physical training [58–61].

### Limitations

This systematic review provides a comprehensive overview of the effect of functional exercise training in pwMS. However, it is important to acknowledge several limitations. Many of the studies included in the analysis were very small and therefore lacked the statistical power to determine efficacy. Beyond that, we reported results on a descriptive basis only due to the heterogeneity of intervention designs and outcomes, and used *p* ≤ 0.05 as a measure of significant effects without providing effect size estimates. Lastly, only two databases were searched which may limit the results retrieved. Additionally, the literature search was based on the herein used definition of functional exercise training. Although our strategy was carefully designed using a combination of MeSH terms and free search terms, our search bears the risk of neglecting studies following divergent definitions and indexing terms related to functional exercise. This limitation applies for example to the studies of Amato et al. [62], Far et al. [63], and Sepehri Far et al. [64] which met the conceptual criteria but were not initially retrieved due to their indexing.

### Future directions

The problem of standardizing the definition and taxonomy of functional training should be the focus of future research, since currently no uniform definition exists. As Ide et al. point out, the term is often used without a clear operational basis [65]. This can result to different training modalities being incorrectly grouped together under the same term. Boyle et al. attempt to provide a practical and goal-oriented definition [17], which was used as a definition in this review. Even though this is a valid point of discussion and an existing limitation, the intention was nevertheless to summarize the current evidence on functional exercise training and their effect in pwMS in a systematic review, considering the numerous potential health benefits, particularly for pwMS. To further substantiate the findings of functional exercise training, future research using similar outcomes is necessary and should focus on improving the consistency of adherence reporting, the homogeneity of the selected assessments tools, and investigate the long-term effects of functional training in the MS population. Additionally, there is a need for more high-quality randomized controlled trials (RCTs) with intentionally designed large sample sizes to achieve statistical significance. These well-powered RCTs are essential for demonstrating predetermined effects or differences accurately.

## Conclusion

Even if the results of the studies included in this review were highly heterogeneous, they suggest that functional training may offer benefits to pwMS. Some of the included studies have demonstrated improvements in functional outcomes (mobility, walking capacity, balance) and physical performance (strength and endurance) because of its ability to target multiple muscle groups simultaneously through more complex multi-joint exercises and thus performing movements that are close to everyday functions. In contrast to other types of training, the specific advantage of functional training for pwMS is its comprehensive approach to achieving functional improvements, strength gains, and improved endurance simultaneously. Further results of high-quality studies could serve as basis for practicing physiotherapists and sports therapists to develop individualized functional exercise regimens for pwMS, and may empower pwMS to engage in functional exercise training without the need for large equipment or gym access, thereby overcoming the physical activity barriers frequently reported by pwMS.

## Supplementary Information

Below is the link to the electronic supplementary material.

Supplementary file1 (DOCX 67 KB)

Supplementary file2 (DOCX 332 KB)

## Data Availability

The data that support the findings of this study are available on request from the corresponding author, NJ.
