# Supplementary material for: Functional exercise training in persons with multiple sclerosis: a systematic review
Source: J Neurol. 2025 Aug 23;272(9):590. doi: 10.1007/s00415-025-13311-w (PMC12374876; doi:10.1007/s00415-025-13311-w)
Supplement: Supplementary file 1 — Supplementary file1 (DOCX 67 KB) [file 415_2025_13311_MOESM1_ESM.docx]

**Supplementary Material 1**

**Journal:** Journal of Neurology

**Title:** Functional exercise training in persons with multiple sclerosis: a systematic review

**Authors:** Frederike Adammek^1,4†^, Weronika Gralla^2,5†^, Marie Kupjetz^1^, Annette Rademacher^3^, Philipp Zimmer^1^, Eduard Isenmann^2^, Niklas Joisten^1*^

^1^Research Group "Sports Medicine", Institute for Sport and Sport Science, TU Dortmund University, Dortmund, 44227, Germany

^2^Department of Fitness and Health, IST University of Applied Sciences, Düsseldorf, 40233, Germany

^3^Asklepios MVZ Bayern GmbH, Cham, 93413, Germany

^4^Department of Neurology, Clinics of Valens, Rehabilitation Centre Valens, Valens, 7317, Switzerland

^5^St Josef-Hospital, Bochum, 44791, Germany

**^†^**FA and WG share first authorship

*Corresponding author. *E-mail address*: [niklas.joisten@tu-dortmund.de](mailto:niklas.joisten@tu-dortmund.de) (N. Joisten)

**Table 3**

Functional exercise training studies in persons with multiple sclerosis (extended version)

|  | Design | Sample  size | Sex  [n (%) female] | Age  [yrs] | EDSS | TSD [yrs] | MS type | Frequency | Intensity | Time | Type | Control | Outcomes | Between-group | Within-  group | |
| --- | --- | --- | --- | --- | --- | --- | --- | --- | --- | --- | --- | --- | --- | --- | --- | --- |
| Abbaspoor et al., 2020 | RCT | INT: 10  CON: 10 | 20 (100) | 33.50 ± 6.37  36.75 ± 6.80 | 3.06 ± 1.20  3.00 ± 1.10 | 10.25 ± 3.37  10.00 ± 3.85 | RRMS: 20 (100) | 3x/week  8 weeks | RPE  10-16  55-70% HR_max_ | 5 exercises;  1-2 sets x 8-14 reps; 60-120 s rest  15-20 min | **WB resistance**  BW; elastic bands;  TRX^®^ suspension  Rhythmic AT | Habitual lifestyle | Serum markers (BDNF, IGF-1); walking speed (10MWT); walking endurance (2MWT); quadriceps strength (dynamometry); handgrip/finger strength (dynamometry) | INT: IGF-1 ↑;  10MWT ↑;  Handgrip strength (R) ↑;  BDNF ↔; 2MWT ↔; Handgrip strength (L) ↔; Finger strength (R/L) ↔ | INT:  2MWT ↑; Quadriceps strength ↑; Handgrip strength (L) ↑;  CON: Finger strength (R/L) ↑ | |
| Akbar et al., 2020 | CT | INT1: 5  INT2: 5 | 5 (100)  4 (80) | 45.6 ± 12.8  53.0 ± 10.7 | N/A | 10.20 ± 6.83  18.60 ± 5.98 | RRMS: 5 (100);SPMS: 0 (0); PPMS 0 (0);  INT2: RRMS: 3 (60); SPMS: 1 (20); PPMS 1 (20); | 3x/week  16 weeks | N/A | 10 exercises, 1-3 sets x 10-15 reps | **WB resistance**  BW; elastic bands; weighted vests  Stretching | INT2: Stretching | Fatigue (Modified Fatigue Impact Scale (total, physical, cognitive, psychosocial); physical activity (Godin Leisure-Time Exercise Questionnaire); grip strength (dynamometry); brain connectivity (resting-state functional MRI) | INT1 vs. INT2: Functional connectivity caudate and inferior parietal region (L), inferior frontal region (R/L), middle frontal region (L), insula (R) ↕; modified Fatigue Impact Scale (total, physical, cognitive, psychosocial) ↔; Godin Leisure-Time Exercise Questionnaire ↔; grip strength | – | |
| Ayán Pérez et al., 2007 | UCT | INT: 24 (36)^1^ | 15 (63) | 44.4 ± 9.5 | 1.5 ± N/A | 8.17 ± 8.3 | SPMS: 24 (100) | 3x/week  6 weeks | Self-chosen  (2-3 reps less than subjective max) | 12 exercises; 1-2 sets x individual number of reps; ~ 2min medicine ball throws;  60 min | **Core strength & WB mobility**  BW; calisthenics; medicine balls | - | Walking speed (9 m zig-zag run);  mobility of arms and time-space orientation (clapping test, dynamic flexibility test); explosiveness of arms (overhead medicine ball throw); explosiveness of legs (vertical jump); trunk strength (abdominal test, back muscle test) leg lifts, Kraus-Weber test); Balance (flamingo balance test) | - | Overhead medicine ball throw ↑; clapping test ↑; dynamic flexibility test ↑; 9 m zig-zag run ↑;  leg lifts ↑, back muscle test ↑; abdominal test ↑ | |
| Bilek et al., 2022 | RCT | INT: 30  CON: 30 | 48 (86)* | 28.29 ± 6.57  31.36 ± 8.07 | 1.71 ± 1.13  2.00 ± 1.14 | 7.46 ± 4.04  9.21 ± 4.29 | RRMS: 60 (100) | 3x/week  8 weeks | N/A  70% V̇O_2peak_ | 60-70 min;  10 exercises;  1-3 sets x 10-15 reps; rest N/A  30 min | **WB resistance**  Elastic bands;  weighted vests  AT (bicycle) | Habitual lifestyle | Cardioresp. fitness (V̇O_2 peak_); serum markers (Contactin-1, Contaction-2);  Cognition (PASAT-3) | INT vs. CON: V̇O_2 peak_ ↑; Contactin-1 ↑; Contaction-2 ↑; PASAT-3 ↑ | INT: V̇O_2 peak_ ↑; Contactin-1 ↑; Contaction-2 ↑; PASAT-3 ↑ | |
| Cakt et al., 2010 | RCT | INT1: 10  INT2:14  CON: 9 | 8 (80)  9 (64)  6 (67) | 43.0 ± 10.2  36.4 ± 10.5  35.5 ± 10.9 | N/A | 6.2 ± 2.2  9.2 ± 5.0  6.6 ± 2.4 | RRMS: N/A; SPMS: N/A | 2x/week  8 weeks | 40% total maximum workload | 15-25 min LB plyometrics + balance; 2 exercises LB plyometrics  15 sets x 2 min high-resistance cycling, 2 min low-resistance/rest | **INT1: LB plyometrics**  INT 1 : Balance exercise  Walking, standing, balance board  INT 2: Additional High-resistance cycling  Bicycle ergometer | Habitual lifestyle | Mobility (TUG); dynamic balance (Dynamic Gait Index); static balance (Functional Reach); fear of falling (Falls Efficacy Scale); walking speed (10MWT); fatigue (Fatigue Severity Scale); depression (Beck Depression Inventory); HRQoL (Short Form-36); total maximum workload; exercise duration | INT2 vs. INT 1 vs. CON: TUG ↑; Dynamic Gait Index ↑; Functional Reach ↑; Falls Efficacy Scale ↑; Fatigue Severity Scale ↑; Beck Depression Inventory ↑; total maximum workload ↑; exercise duration ↑; 10MWT ↔; SF-36 ↔;  INT2 vs. CON: 10MWT ↑ | INT1: Falls Efficacy Scale ↑; total maximum workload ↑; exercise duration ↑; Short Form-36 (physical functioning ↑; TUG ↔; Dynamic Gait Index ↔; Functional Reach ↔; 10MWT ↔; Fatigue Severity Scale ↔; Beck Depression Inventory ↔;  INT2: TUG ↑; Dynamic Gait Index ↑; Functional Reach ↑; Falls Efficacy Scale ↑; 10MWT ↑; Fatigue Severity Scale ↑; Beck Depression Inventory ↑; Short Form-36 (physical functioning, role-physical functioning) ↑; total maximum workload ↑; exercise duration  ↑;  CON: all outcomes ↔ | |
| Coote et al., 2015 | RCT | INT1: 10  INT2: 15 | 6 (60)  11 (73) | 51.8 ± 12.1  51.8 ± 12.6 | N/A | 12.2 ± 4  11.8 ± 5.5 | RRMS: 4 (40); SPMS: 2 (20); PPMS 3 (30); N/A: 1 (10);  INT2: RRMS: 8 (53); SPMS: 1 (7); PPMS 4 (27); BMS 1 (7); N/A: 1 (7) | 2x/week  6 weeks; 3x/week  6 weeks | Adj. until muscular failure | 6 exercises; 1-3 sets x 12 reps, 2-3 min rest | **INT1: LB resistance**  BW, wooden box, free weights (hand, ankle, backpack)  INT2: LB resistance combined with neuromuscular electric stimulation  s.a | INT2: LB combined with neuromuscular electric stimulation | Knee extension strength (dynamometry); hip extension strength (dynamometry); lower extremity strength (maximum rep Sit To Stand Test); lower-limb spasticity (VAS); mobility (TUG); self-reported walking impairment (MS Walking Scale–12); balance (Berg Balance Scale); impact of MS (MS Impact Scale-29); fatigue (Modified Fatigue Impact Scale) | INT1 vs. INT2: Modified Fatigue Impact Scale (total) ↓; hip/knee extension strength ↔; Sit To Stand ↔; spasticity VAS ↔; TUG ↔; MS Walking Scale-12 ↔; MS Impact Scale (physiological/psychological score) ↔; Berg Balance Scale ↔; | INT1: Hip extension strength ↑; knee extension strength ↔; Sit To Stand ↔; spasticity VAS ↔; TUG ↔; MS Walking Scale-12 ↔; MS Impact Scale (physiological/ psychological) ↔; Modified Fatigue Impact Scale (total) ↔;  INT2: Hip extension strength ↑; Sit To Stand ↑; Berg Balance Scale ↑; MS Impact Scale (physiological) ↑; Modified Fatigue Impact Scale (total) ↑; knee extension strength ↔; spasticity VAS ↔; TUG ↔; MS Walking Scale-12 ↔; MS Impact Scale (psychological) ↔ | |
| Correale et al., 2021 | RCT | INT: 14  CON: 13 | 27 (100) | 45.4 ± 7.2  48.3 ± 6.1 | 2.25 ± 0.8 | 14.6 ± 6.9 | RRMS:  27 (100) | 2x/week  12 weeks | Self-chosen | 45-60 min;  3 sets x 8-12 reps; 60-90 sec rest  25 min; 50-70% HRR | **WB resistance**  Calisthenics; dumbells; elastic bands  AT (bicycle/treadmill) | N/A | Strength quadriceps (MVIC, 1-RM leg extension); strength chest (1-RM chest press); strength back (1-RM seated row); fatigue (MFIS); depressive symptoms (BDI-II); HRQoL (MSQOL-54) | INT vs. CON: MFIS ↑; MSQOL-54 (mental composite) ↑;  MVIC ↔; 1-RM leg extension ↔; 1-RM chest press ↔; 1-RM seated row ↔; depressive symptoms ↔; MSQOL-54 (physical composite) ↔ | INT: MVIC ↑; 1-RM leg extension ↑; 1-RM chest press ↑; MSQOL-54 (physical and mental composite) ↑; depressive symptoms ↑; 1-RM seated row ↔; INT (post - after 12 wks detraining): MFIS ↓; MVIC ↔, 1-RM leg extension ↔; 1-RM chest press ↔; 1-RM, seated row ↔; depressive symptoms ↔; MSQOL-54 (physical and mental composite) ↔;  CON: depressive symptoms ↑, all other outcomes ↔ | |
| De Bolt and McCubbin, 2004 | RCT | INT: 19  CON: 18 | 15 (79)  14 (78) | 51.63 ± 6.71;  47.78 ± 10.47 | 3.97 ± 1.79;  3.50 ± 1.50 | 15.05 ± 12.23  13.08 ± 11.21 | INT: RRMS 9 (47); PMS 9 (47); BMS 1 (5);  CON: RRMS 8 (44); PMS 9 (50); BMS 1 (6) | 3x/week  8 weeks | ≥ 0.5% BW | 25-30 min, 2-3 sets x 8-12 reps; self-chosen rests | **LB resistance**  BW, weighted vests, ankle weights, step | Habitual lifestyle | Balance (antero-posterior sway, mediolateral sway, sway velocity); leg extensor power (maximal leg extension power); mobility (TUG) | INT vs. CON: absolute/relative leg extensor power ↑; anteroposterior/mediolateral sway ↔; sway velocity ↔; TUG ↔ | INT: absolute/relative leg extensor power ↑; anteroposterior/mediolateral sway ↔; sway velocity ↔; TUG ↔;  CON: all outcomes ↔ | |
| Frevel and Mäurer, 2015 | RCT | INT1: 9  INT2: 9 | 7 (78)  8 (89) | 44.3 ± 8.1;  46.9 ± 7.6 | 3.8 ± 1.5;  3.8 ± 1.1 | 16.1 ± 11.3;  22.3 ± 8.3 | INT1: RRMS 6 (67); SPMS 3 (33);  INT2: RRMS 6 (67); SPMS 3 (33) | 2x/week  12 weeks | RPE 11-14 | 45 min; 5-8 exercises; 2-3 sets x 8-15 reps  20-30 min | **INT1: LB resistance**  BW, elastic bands, gym ball, mat  INT1: Balance  Standing, unstable surface  INT2: Hippotherapy | INT2: Hippotherapy | Balance (Berg Balance Scale; Dynamic Gait Index); knee extensor/flexor strength (MVIC); trunk extensor/flexor strength (MVIC); mobility (TUG); walking speed (2MWT 1 minute/total); HRQoL (Hamburg Quality of Life Questionnaire in Multiple Sclerosis (HAQUAMS, total, all subscales); fatigue (Fatigue Severity Scale; Modified Fatigue Impact Scale) | INT vs. CON: MFIS cognitive subscale ↑; HAQUAMS upper limb ↑; Berg Balance Scale ↔; Dynamic Gait Index ↔; knee extensor/flexor MVIC ↔; trunk extensor/flexor MVIC ↔ | INT1: Berg Balance Scale ↑; Dynamic Gait Index ↑; MFIS cognitive subscale ↑; knee extensor/flexor MVIC ↔; trunk extensor/flexor MVIC ↔; 2MWT (1 min, total) ↔; TUG ↔; Fatigue Severity Scale ↔; HAQUAMS ↔;  INT2: Berg Balance Scale ↑; Dynamic Gait Index ↑; 2MWT (1 minute) ↑; MFIS (total, all subscales) ↑; Fatigue Severity Scale ↑; HAQUAMS (cognitive function, lower limb function, mood) ↑; knee extensor/flexor MVIC ↔; trunk extensor/flexor MVIC ↔; TUG ↔; 2MWT total ↔ | |
| Garrett et al., 2013 | RCT | INT1: 63^#^;  INT2: 67^#^;  INT3: 63^#^;  CON: 49^#^ | 50 (79);  45 (68); 44 (70);  43 (87) | 51.7 ± 10; 50.3 ± 10; 49.6 ± 10; 48.8 ± 11 | N/A | INT1: 9.8 ± 7.0; INT2: 10.5 ± 6.9; INT3: 11.6 ± 8.0; CON: 10.6 ± 8.2 | INT1: RRMS 35 (55); SPMS 9 (14); PPMS 5 (7); N/A 14 (22); INT2: RRMS 33 (49); SPMS 13 (19); PPMS 9 (13); BMS 3 (5); N/A 9 (13);  INT3: RRMS 38 (60); SPMS 7 (11); PPMS 8 (13); BMS 1 (2); N/A 9 (1);  CON: RRMS 27 (55); SPMS 10 (20); PPMS 3 (6); BMS 1 (2); N/A 8 (16) | 1x/week  10 weeks  2x/week  10 weeks  1x/week  10 weeks  1x/week  10 weeks | Until muscular failure  65% HR_max_, RPE 11-14  Not predefined  Not predefined | 60 min; 3 sets x 12 reps  30 min  30 min  Not predefined  Not predefined | **INT1: WB resistance**  BW, free weights  INT1: AT  Mode of choice (walking/cycling/swimming/running)  INT2: Resistance training/AT/combined training  Not predefined  INT3: Yoga  Not predefined | INT2: Resistance/AT/combined training  INT3: Yoga  CON: Habitual lifestyle | HRQoL (Multiple Sclerosis Impact  Scale-29 (physical/psychological)); fatigue (Modified Fatigue Impact Scale ( total/physical/cognitive)); walking endurance (6MWT) | INT1 vs. CON: Multiple Sclerosis Impact  Scale-29 (physical/psychological) ↑; Modified Fatigue Impact Scale (total/physical) ↑; Modified Fatigue Impact Scale (cognitive) ↔;  6MWT ↑  INT2 vs. CON: Multiple Sclerosis Impact  Scale-29 (physical/psychological) ↑; Modified Fatigue Impact Scale (total/physical) ↑; Modified Fatigue Impact Scale (cognitive) ↔ ; 6MWT ↑  INT3 vs. CON: Multiple Sclerosis Impact  Scale-29 (psychological) ↑; Modified Fatigue Impact Scale (total/physical) ↑; Multiple Sclerosis Impact  Scale-29 (physical) ↔; Modified Fatigue Impact Scale (cognitive) ↔;  6MWT ↔ | INT1: Multiple Sclerosis Impact  Scale-29 (physical/psychological) ↑; Modified Fatigue Impact Scale ( total/physical/cognitive) ↑; 6MWT ↑  INT2: Multiple Sclerosis Impact  Scale-29 (physical/psychological) ↑; Modified Fatigue Impact Scale (total/physical/cognitive) ↑; 6MWT ↑  INT3: Multiple Sclerosis Impact  Scale-29 (physical/psychological) ↑; Modified Fatigue Impact Scale (total/physical/cognitive) ↑; 6MWT ↔  CON: all outcomes ↔ | |
| Hosseini et al., 2018 | RCT | INT1: 9  INT2: 9  CON: 8 | 5 (56)  5 (56)  4 (50) | 32.9 ± 8.1;  31.3 ± 7.1;  33.0 ± 9.7 | N/A | N/A | N/A | 3x/week  8 weeks | ≥ 1% bodyweight | 5 exercises; 3 sets x 10 reps, 30-60 s rest (betw. exercises)  60-70 min; 1 set x 30-60 s, 30-60 s rest | **INT1: LB resistance**  Free weights (fastened to the body)  INT2: Hatha Yoga  Standing, sitting, lying | Habitual lifestyle | Leg extensor strength (1-RM); leg strength (leg press); walking speed (10MWT); balance (postural sway balance index, two legs open/closed eyes, one leg open eyes) | INT1 vs. INT2 vs. CON : Leg extensor 1-RM ↕ (INT1 ↑) | INT1: Leg extensor 1-RM ↑; 10MWT ↑; postural sway balance index (two legs closed eyes) ↑; postural sway balance index (two legs/one leg open eyes) ↔;  INT2: postural sway balance index (one leg/two legs open eyes) ↑; leg extensor 1-RM ↔; 10MWT ↔; postural sway balance index (two legs closed eyes) ↔; CON: postural sway balance index (two legs open eyes): ↑, all other outcomes ↔ | |
| Learmonthet al., 2012 | RCT | INT: 20  CON: 12 | 15 (67)  8 (67) | 51.4 ± 8.06  51.8 ± 8.0 | 6.14 ± 0.36  5.82 ± 0.51 | 13.4 ± 6.4  12.6 ± 8.1 | N/A | 2x/week  12 weeks | Self-chosen | 30-40 min;  8-12 exercises; N/A sets x 1 min;  ≥ 60 s rest | **WB resistance**  BW,dumbells, chair  AT  Walking/stepping/bike/Foot pedals  Balance  Sitting/standing/walking | Habitual lifestyle | Walking speed (T25FW); body mass index; walking endurance (6MWT); balance (Berg Balance Scale); mobility (TUG); quadriceps strength (maximum isometric force leg extension, weaker leg); activity level (PhoneFITT); balance confidence (Activities Balance Confidence); fatigue (Fatigue Severity Scale); anxiety and depressive symptoms (HADS); HRQoL (LMSQOL); goal attainment (Goal Attainment Scale) | T25FW ↔; PhoneFITT ↕; Activities Balance Confidence ↑ (0-12 wks); body mass index ↔; 6MWT ↔; Berg Balance Scale ↔; TUG ↔; maximum isometric force leg extension ↔; fatigue severity scale ↔; HADS ↔; LMSQOL ↔ | No significant effects. | |
| Mardaniyan Ghahfarrokhi et al., 2022 | RCT | INT1: 15;  INT2: 15 | 12 (80),  13(87) | 39.87 ± 9.09;  37.50 ± 8.58 | 4.13 ± 0.97;  4.57 ± 1.30 | 9.73 ± 5.29;  8.30 ± 6.48 | INT1: RRMS 15 (100);  INT2: RRMS 15 (100) | 3x/week  8 weeks | CR-10 :  2-6  CR-10 :  2-6 | 90-120 min, 14 exercises, 1-2 sets x 10-12 reps  90-120 min, 2 sets x 8 reps, 2 min rest | **INT1: WB resistance**  Elastic bands  **INT2: Neurofunctional exercise**  Balance (standing, walking), walking, WB resistance, pelvic control, core stability, BW, cones, Swissball | INT2: Neurofunctional training | Static balance (wide/narrow, single-leg (R/L, open/closed eyes), semi-tandem, tandem open/closed eyes); dynamic balance (tandem walk test); mobility (TUG); walking endurance (6MWT); walking speed (10MWT, T25FWT);  walking ability (6 Spot Step Test (dominant/non-dominant); lower extremity strength (5 Times Sit to Stand); hand grip strength (dynamometry) | INT1 vs. INT2: Tandem walk ↓; Six Spot Step Test (non-dominant) ↓; 6MWT ↓; all other outcomes ↔ | INT1: 6MWT ↑; 5 Times Sit to Stand ↑; all other outcomes ↔  INT2: Tandem walk ↑; Six Spot Step Test (non-dominant) ↑; 6MWT ↑; 5 Times Sit to Stand ↑; all other outcomes ↔ | |
| Moghadasi et al., 2020 | CT | INT: 19  CON: 15 | 34 (100) | 37.62 ± 4.58,  34.72 ± 5.01 | 2.18 ± 0.85,  2.59 ± 1.01 | 8.28 ± 3.90,  7.09 ± 3.38 | RRMS:  34 (100) | 3x/week  8 weeks | N/A | 30min; 8 exercises; 3 sets x 5-10 reps | **WB Resistance**  TRX^®^ suspension | Habitual lifestyle | Mobility (TUG); walking speed (10MWT); walking endurance (2MWT); lower extremity strength (5 Times Sit to Stand); joint position sense (knee proprioception absolute error); quadriceps/knee extensor strength (MVIC); knee flexor strength (MVIC) | INT vs. CON: TUG ↑; 10MWT ↑; 2MWT ↑; 5 Times Sit to Stand ↑; knee proprioception absolute error non-dominant leg (60°) ↑ ;MVIC knee extensors dominant/non-dominant (20° and 70°) ↑; MVIC knee flexors dominant/non-dominant (20°) ↑; MVIC knee flexors dominant (70°) ↑; knee proprioception absolute error dominant leg (60°) ↔; proprioception absolute error dominant and non-dominant leg (30°) ↔; MVIC knee flexors non-dominant (70°) ↔ | – | |
| Motl et al., 2012 | UCT | INT: 13 | 8 (62) | 51.5 ± 11.3 | 5.58 ± 0.79 | 11.2 ± 6.9 | RRMS: 11 (85);  SPMS: 1 (8); PPMS 1 (8) | 3x/week  8 weeks | RPE 13  RPE 13  RPE 13 | 15-60 min;  5-20 min; 5 exercises; sets/reps N/A  5-20 min  5-20 min | **LB resistance**  Elastic bands  AT  Ergometry (bicycle, rowing, elliptical)/treadmill  Balance  Standing, walking | – | Walking speed (T25FW); self-reported walking impairment (MS Walking Scale-12); mobility (TUG); gait efficiency (Functional Gait Profile score, spatial and temporal gait measures) | – | MS Walking Scale–12 ↑; T25FW ↑; TUG ↑; Functional Gait Profile score ↑; gait velocity ↑; stride length ↑; single support ↑; swing phase ↑; cadence ↔; step time ↔; base of support ↔; double support ↔ | |
| Najafi et al., 2019 | RCT | INT: 30  CON: 30 | 60 (100) | 38.39 ± 4.59  36.36 ± 3.54 | 2.51 ± 1.22  2.44 ± 0.77 | N/A | RRMS 60 (100) | 3x/week  8 weeks | N/A | 45-60 min; 5 exercises; 3-4 sets x 10-15 s, 10-15 s rest | **Core training**  BW  **Balance**  Standing, walking (open/closed eyes) | N/A | Postural control (Center of Pressure sway area and path length, opened/closed eyes); balance (Berg Balance Scale); walking speed (T25FW); mobility (TUG) | Center of Pressure sway area (opened/closed eyes) ↑; Center of Pressure path length (opened/closed eyes) ↑; Berg Balance Scale ↑; T25FW ↑; TUG ↑ | – |  |
| Sabapathy et al., 2011 | RCT | INT1: 11 (14)^1^  INT2: 5 (6)^1^ | 12 (75) | 55 ± 7 | N/A | 10 ± 10 | RRMS 10 (63); SPMS 3 (19); PPMS 3 (19) | 2x/week  8 weeks | CR10 3-5 | 8 exercises, 2-3 sets x 6-10 reps, ≥ 30-60 s rest  15-20 min  8 stations x 5 min, 2 min rest every 10 min  15-20 min | **INT1: WB resistance and balance**  Elastic band, dumbell (1-4kg), parallel bar, Swiss ball, ankle weights (1-5kg), foam mat, foam beam, wobble board  **INT1: Stretching**  INT2: AT  Arm crank, cycling, cross-trainer, step-ups, recumbent cycling, treadmill  INT2: Stretching | INT2: AT | Grip strength (dynamometry); static balance (functional reach), dynamic balance (4 Step Square); mobility (TUG); walking endurance (6MWT); HRQoL (Multiple Sclerosis Impact Scale (physical/psychological); Short Form-36 (Physical and Mental Component Summary); fatigue (Modified Fatigue Impact Scale (physical/cognitive/psychosocial)); depressive symptoms (Becks Depression Inventory) | INT1 vs. INT 2: all outcomes ↔ | INT1: Functional Reach ↑ ; 4 Step Square ↑; TUG ↑; 6MWT ↑; Multiple Sclerosis Impact Scale (physical) ↑; Modified Fatigue Impact Scale (physical/psychosocial) ↑; grip strength ↔; Multiple Sclerosis Impact Scale (psychological) ↔; Modified Fatigue Impact Scale (cognitive) ↔; Becks Depression Inventory ↔; Short Form-36 (Physical and Mental Component Summary) ↔  INT2: Functional Reach ↑ ; 4 Step Square ↑; TUG ↑; 6MWT ↑; Multiple Sclerosis Impact Scale (physical) ↑; Modified Fatigue Impact Scale (physical/psychosocial) ↑; grip strength ↔; Multiple Sclerosis Impact Scale (psychological) ↔; Modified Fatigue Impact Scale (cognitive) ↔; Becks Depression Inventory ↔; Short Form-36 (Physical and Mental Component Summary) ↔ |  |
| Sosnoff et al., 2014 | RCT | INT: 13  CON:  14 | 10 (77),  11 (79) | 60.1 ± 6.3,  60.1 ± 6.0 | 5.5 (2.5),  5.5 (3.5)  [Mdn (IQR)] | 13.9 ± 6.7,  17.7 ± 11.3 | INT: RRMS: 10 (77); SPMS: 1 (8); PPMS: 2 (15);  CON: RRMS: 10 (71), SPMS: 3 (21) ; PPMS: 1 (7) | 3x/week  12 weeks | N/A | 45-60 min;  3 sets x 8-12 reps | **LB and core resistance**  Elastic bands, BW  Balance  Standing, walking | Habitual lifestyle | Fall risk (Physiological Profile Assessment total, subcomponents); balance (Berg Balance Scale); balance confidence (Activities-  Specific Balance Confidence scale); walking speed (T25FW); walking endurance (6MWT); mobility (TUG); self-reported walking impairment (MS Walking Scale-12); self-reported fall frequency | INC vs. CON: Physiological Profile Assessment total ↑; postural sway subcomponent ↑; T25FW ↑; Activities-  Specific Balance Confidence scale ↑; other subcomponents (Melbourne Edge detection test, proprioception, strength, reaction time) ↔; 6MWT ↔; TUG ↔; MS Walking Scale-12 ↔; Berg Balance Scale ↔ | – |  |
| Zaenker et al., 2018 | UCT | INT: 26 (30)^1^ | 19 (73) | 44.6 ± 7.9 | 2.46 ± 1.52 | 12.3 ± 7.6 | RRMS: 22 (85); SPMS: 3 (12), PPMS; 1 (4) | 2x/week  1-4 week  3x/week  5-12 week | N/A  Intervals at 90-110% maximal tolerated power, rest at anaerobic threshold  moderate | 4 exercises, 4-5 sets x 10-15 reps  s.a  17 min  35-45 min | **LB resistance**  BW, resistance band, ankle weight  **Additional LB** **resistance** (5-12 week, every 2^nd^ week)  AT  HIIT: 5 intervals, 3 min rest  Additional AT (5-12 week, every 2^nd^ week) Self-chosen | – | Cardiorespiratory fitness (VO_2 peak_, lactate at the end of the maximum aerobic test, HR_peak_), physical capacity (maximal tolerated power), quadriceps strength (isokinetic peak torque), hamstring strength (isokinetic peak torque); HRQoL (MSQOL-54 total, subscales) | – | VO_2 peak_ ↑; maximum tolerated power ↑; lactate at the end of the maximum aerobic test ↑, HR_max_ ↑, quadriceps isokinetic peak torque (R/L) ↑; hamstring isokinetic peak torque (R/L) ↑; MSQOL-54 (vitality, emotional well-being, general well-being) ↑ |  |

Notes: intervention duration without warm-up/cool-down periods; *of participants analyzed; ^#^whole group prior drop-out; bold = in multicomponent interventions considered as “functional training”, ↔ = no change, ↑ = significant improvement/between-group effect in favor of INT(1); ↓ = significant worsening/between-group in favor of INT2/CON; ↕ = unspecified significant difference, ^1^Data presented for n completing the program, initial sample size in brackets. Abbreviations: AT = Aerobic training; BDNF = brain-derived neurotrophic factor; BDI-II = Beck Depression Inventory-II; BMS = benign MS; CPET = cardiopulmonary exercise test; CON = control group; CT = controlled trial; EDSS = Expanded Disability Status Scale; f = female; HRR = heart rate reserve; HADS = Hospital Anxiety and Depression Scale; HAQUAMS = Hamburg Quality of Life Questionnaire in Multiple Sclerosis; HR_max_ / HR_peak_= maximum /peak heart rate; HRQoL = Health-related quality of life; IGF-1 = insulin like growth factor ; INT = intervention group; L = leftLB = lower body; m = male; LMSQOL = Leeds Multiple Sclerosis Quality of Life Scale; MFIS = Modified Fatigue Impact Scale; MRI = Magnetic resonance imaging; MSQOL-54 = Multiple Sclerosis Quality of Life instrument-54 item; MVIC = maximal voluntary isometric contraction; PASAT-3 = Paced Auditory Serial Addition Test with 3 s stimulus; PMS = progressive MS; PPMS = primary progressive MS; PSFS = Patient-Specific Functional Scale; R = right; RCT = randomized controlled trial; RPE = Rate of Perceived Exertion; RRMS = relapsing-remitting MS; RT = resistance training; SF-36 = Short Form-36; SPMS = secondary progressive MS; TUG = Timed Up and Go; TSD = time since diagnosis; T25FW = Timed 25-Foot Walk; UCT = uncontrolled trial; VAS = visual analog scale; V̇O_2 max_ / V̇O_2 peak_ = maximum/peak oxygen consumption; WB = whole body; 1-RM = 1-Repition Maximum; 2MWT = 2-Minute Walk Test; 6MWT = 6-Minute Walk Test; 10MWT = 10 Meter Walk Test.
