# Supplementary material for: Functional exercise training in persons with multiple sclerosis: a systematic review
Source: J Neurol. 2025 Aug 23;272(9):590. doi: 10.1007/s00415-025-13311-w (PMC12374876; doi:10.1007/s00415-025-13311-w)
Supplement: Supplementary file 2 — Supplementary file2 (DOCX 332 KB) [file 415_2025_13311_MOESM2_ESM.docx]

**Supplementary Material 2**

**Journal:** Journal of Neurology

**Title:** Functional exercise training in persons with multiple sclerosis: a systematic review

**Authors:** Frederike Adammek^1,4†^, Weronika Gralla^2,5†^, Marie Kupjetz^1^, Annette Rademacher^3^, Philipp Zimmer^1^, Eduard Isenmann^2^, Niklas Joisten^1*^

^1^Research Group "Sports Medicine", Institute for Sport and Sport Science, TU Dortmund University, Dortmund, 44227, Germany

^2^Department of Fitness and Health, IST University of Applied Sciences, Düsseldorf, 40233, Germany

^3^Asklepios MVZ Bayern GmbH, Cham, 93413, Germany

^4^Department of Neurology, Clinics of Valens, Rehabilitation Centre Valens, Valens, 7317, Switzerland

^5^St Josef-Hospital, Bochum, 44791, Germany

**^†^**FA and WG share first authorship

*Corresponding author. *E-mail address*: [niklas.joisten@tu-dortmund.de](mailto:niklas.joisten@tu-dortmund.de) (N. Joisten)

**Risk of bias of the included studies**

The assessment of the 19 studies included in this review showed that the risk of bias was rated as high or with some concern in several domains, particularly with regard to outcome reporting (domain 5) and deviations from the intended intervention (domain 2).

The measurement of outcomes (domain 4) was predominantly rated as uncritical.

**
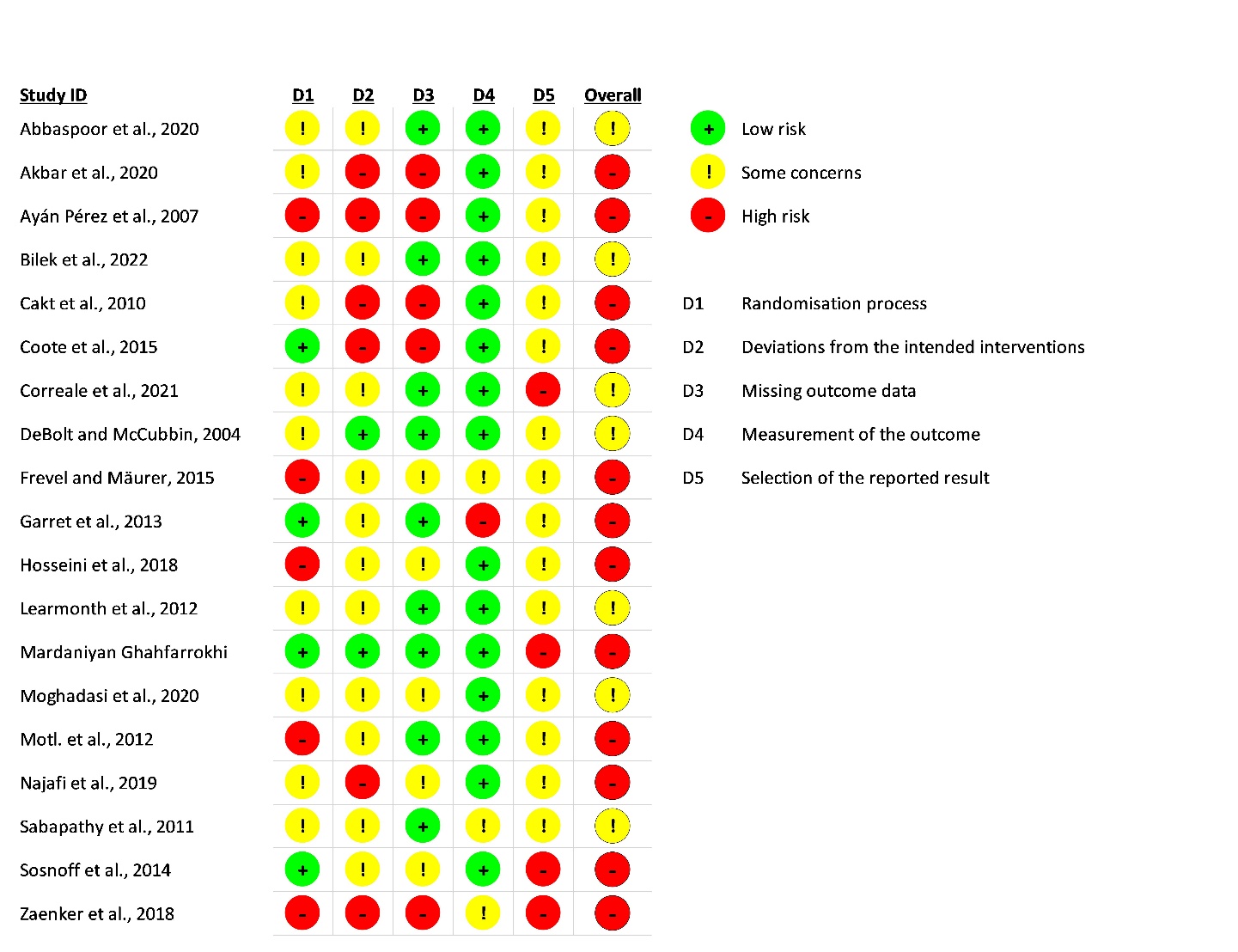
**
